# Neonatal loss of FGFR2 in astroglial cells affects locomotion, sociability, working memory, and glia-neuron interactions in mice

**DOI:** 10.1038/s41398-023-02372-y

**Published:** 2023-03-11

**Authors:** Hanna E. Stevens, Soraya Scuderi, Sarah C. Collica, Simone Tomasi, Tamas L. Horvath, Flora M. Vaccarino

**Affiliations:** 1 Child Study Center, Yale School of Medicine, New Haven, CT 06520 USA; 2 Department of Psychiatry, Department of Pediatrics, University of Iowa Carver College of Medicine, Iowa City, IA 52246 USA; 3 Department of Neuroscience, Yale University, New Haven, CT 06520 USA; 4 Department of Comparative Medicine, Department of Obstetrics and Gynecology, Yale School of Medicine, New Haven, CT 06520 USA

**Keywords:** Molecular neuroscience, ADHD

## Abstract

Fibroblast growth factor receptor 2 (FGFR2) is almost exclusively expressed in glial cells in postnatal mouse brain, but its impact in glia for brain behavioral functioning is poorly understood. We compared behavioral effects from FGFR2 loss in both neurons and astroglial cells and from FGFR2 loss in astroglial cells by using either the pluripotent progenitor-driven *hGFAP-cre* or the tamoxifen-inducible astrocyte-driven *GFAP-creER*^*T2*^ in *Fgfr2* floxed mice. When FGFR2 was eliminated in embryonic pluripotent precursors or in early postnatal astroglia, mice were hyperactive, and had small changes in working memory, sociability, and anxiety-like behavior. In contrast, FGFR2 loss in astrocytes starting at 8 weeks of age resulted only in reduced anxiety-like behavior. Therefore, early postnatal loss of FGFR2 in astroglia is critical for broad behavioral dysregulation. Neurobiological assessments demonstrated that astrocyte-neuron membrane contact was reduced and glial glutamine synthetase expression increased only by early postnatal FGFR2 loss. We conclude that altered astroglial cell function dependent on FGFR2 in the early postnatal period may result in impaired synaptic development and behavioral regulation, modeling childhood behavioral deficits like attention deficit hyperactivity disorder (ADHD).

## Introduction

Fibroblast growth factor (FGF) signaling has been characterized extensively for its role in the brain, particularly in neurons and their precursors [1–4]. We and others have determined the importance of FGF signaling for embryonic and postnatal neurogenesis and cortical expansion [5–8], dendrite and synapse development [9], and expression and responsiveness of neuronal components of the glucocorticoid-response system [10, 11]. Furthermore, changes in locomotor activity, anxiety-like behavior, altered stress response, and social interaction occur in animal models with disrupted FGF ligands or FGF receptor 1 (FGFR1) dependent signaling in the forebrain [3, 10, 12–16], implicating FGF signaling in basic brain development and functioning relevant to psychiatric illness. However, even in these studies, understanding the specific role of FGF receptors and ligands in different cell types for specific behavioral brain functions has been difficult. There are many different interactions of FGF ligands and FGFRs, such that specific roles for FGF ligands—such as FGF17 in social behavior, FGF22 in anhedonia-like behavior, or FGF2 or FGF8 in anxiety-like behavior—leaves undetermined what receptors and cells mediate these effects [10, 14, 17, 18]. This is an important challenge to overcome to understand how FGF signaling may contribute to neuropsychiatric risk [19–22] and potential for treatment [23].

FGF receptors, and in particular FGFR2, are almost exclusively expressed in non-neurons in the postnatal forebrain [24–29]. Although FGF signaling is known to promote astrocyte differentiation and astrocyte activation in the mature brain [30, 31], the role of specific FGFRs in astrocytes is still undefined, and unraveling their separate roles at different developmental stages has been challenging. Evidence has recently arisen through animal model systems that suggests the importance of FGF-signaling alterations in astrocytes for processes of brain development and behavior: specifically, FGFR1 expression in dorsal forebrain astroglia secondarily affects the early postnatal development of cortical interneurons, but impacts of astrocytic FGFR2 during early postnatal development is not known, despite its almost-exclusive presence in astroglia postnatally. In cultured human astrocytes, both FGFR1 and FGFR2 are needed for signal transduction effects of anti-depressants, but FGFR2, not FGFR1, is necessary for acidic-FGF (FGF1) to further stimulate activated astrocytes [24, 32]. Exogenous FGF2 reduces stress-induced changes in astrocytes which may be a pathway to regulation of anxiety-like behavior; [33–35] however, anti-anxiety-like effects of FGF2 in mice do not require FGFR1 or R2 [10]. Many gaps in knowledge about the behavioral impacts of astroglial FGF signaling exist. Specifically, how astrocyte-dependent signaling through FGFR2 affects behavioral regulation across different domains has not been determined, despite its importance in postnatal astroglia.

Astrocyte dysfunction in the forebrain results in mixed behavioral outcomes but suggest a role for astroglia in regulation of activity—specifically disrupted astroglial SynCAMI or adenosine kinase increases locomotor activity [36, 37]. Astrocytes have a critical role in regulating neuronal synaptogenesis and pruning, processes that underlie early learning and behavioral outcomes [38, 39], emphasizing the importance of examining not only behavioral outcomes from FGF effects on astrocyte function but how these relate to synaptic development. There have been calls for greater investigation of astrocytic mechanisms relevant to childhood neurodevelopmental disorders [40, 41].

Here, we specifically targeted FGF signaling in the brain at different times as important mechanisms underlying multiple behaviors by inducing the knockout of FGFR2 at different stages of development and in different cell types. We examined three different sets of mice that had a cell type-targeted knock-out of FGFR2: 1) knockout during embryogenesis, in radial glial cells and therefore both their neuronal and glial progeny 2) knockout during the rodent early postnatal period, largely in proliferating astroglial precursors and glial progeny and 3) knockout during adulthood, in largely post-mitotic astroglia during mature brain functioning. We hypothesized that early postnatal loss of FGFR2 in astrocytes would affect astrocyte biological function and impact the regulation of behaviors that are disrupted at early postnatal time points in psychiatric disorders.

## Materials and method

### Mice

All experimental procedures involving animals were performed in accordance with the Yale University and University of Iowa Animal Resources Center and Institutional Animal Care and Use Committee (IACUC) policies. Sufficient animals or samples were generated for each assessments using a power analysis based on previous studies and α = 0.05 and β = 0.2

Conditional *hGFAP-cre fgfr2* knockout mice (referred to here as cKO) on a mixed background have been previously described [1, 8]. The conditional *fgfr2* null allele harbors loxP recombination sites flanking regions encoding the Ig III binding and transmembrane domains of the *fgfr2* gene (*fgfr2*^*f*^) [42]. Mice homozygous for *fgfr2*^*f*^ alleles were crossed with mice expressing the Cre recombinase transgene under the control of the human *GFAP* promoter (*hGFAP*) [43]. The *hGFAP-cre* transgene targets Cre recombination to radial glia progenitors of the dorsal telencephalon starting at E13.5 [2]. *Cre* negative mice, littermates when possible, were used as control animals.

To assess the contribution of FGFR2 solely in the postnatal brain, mice homozygous for the *fgfr2*^*f*^ alleles were crossed with *GFAP*-*creER*^T2^ (GCE) mice [44] also on a mixed background. The latter express a tamoxifen-inducible Cre recombinase-estrogen receptor fusion protein (CreER^T2^) [45] under the control of the *GFAP* promoter. Specifying gene knock outs to astroglia is challenging due to the overlapping nature of most genetic drivers in both postnatal neural stem cells and astroglia. Therefore, genetic approaches may have impacts on a small number of neural stem cells (which may impact neurons in the dentate gyrus and olfactory bulb) but will effect astroglia much more substantially; our approach must be considered with this caveat. Previous investigations of the GCE line demonstrate that it largely affects astrocytes; [44] in combination with the postnatal expression of FGFR2 in non-neuronal cells, postnatal induction with the GCE line principally targets *fgfr2* loss in astrocytes. We used two different tamoxifen-induction protocols to target the knockout of *fgfr2* at different time points. In the first neonatal protocol (referred to here as nKO), mother mice received intraperitoneal (IP) injections of 1 mg of tamoxifen dissolved in sunflower seed oil once daily for five consecutive days, beginning on postnatal day 1, 2, or 3 while Cre- and Cre+ experimental animals were nursing. In the second adult induction protocol (referred to here as iKO), Cre- and Cre+ adult mice received injections of 0.5 mg of tamoxifen dissolved in sunflower seed oil twice daily for five consecutive days at 2–4 months of age as previously [8]. Behavioral testing began at least 9 days after the time of the last tamoxifen injection. A few control animals with solely sunflower seed oil injection using the neonatal and adult protocols were created and tested on some behavioral assays

### Behavior testing

All behavior assessments were performed during the light cycle in a dedicated testing room with only one behavior assessment performed per day in the order described below, allowing mice to habituate to the testing room for 60 min prior to testing. Unless otherwise noted, mice remained in their home cage with cage-mates immediately before and after assessments. Each of the three types of knockout mice were tested alongside their control littermates. Only male mice were tested due to limited resources; childhood psychiatric disorders have a higher prevalence in males which allows for these data only in males, while limited, to be translationally informative [46]. Behavioral testing in each cohort was performed with mice grouped together for testing across close birthdates, with testing beginning for some mice in each cohort at age 2.5 months and others at 4.5 months of age. All testing was completed when mice were between 5.5 and 7.5 months of age.

Open Field: In a square or rectangular plastic arena, at least 1500 cm^2^ in area, mice were tested for locomotor activity for at least 50 min. Test mice were placed in the corner of the arena and their movements recorded using an overhead camera (Anymaze software; Stoelting Co, Wood Dale, Illinois). The main measure of distance traveled was evaluated in 5 min epochs and repeated measures ANOVA was used to evaluate group differences over time.

Social Approach: In a three-chamber social approach apparatus [47], mice were tested for social recognition. Two male “stranger” mice unfamiliar to the test mouse but of the same strain and age were first habituated for five minutes to small cylinders in each side chamber. Then, test mice were habituated to the center chamber for five minutes. Subsequently, the test mouse underwent a first stage—a ten-minute trial in which only one stranger was present in one side chamber. This was followed by a second stage—a ten minute trial in which the previous stranger mouse and a new stranger mouse were present in the two side chambers. Movement was recorded using an overhead camera and evaluated for the amount of time the test mouse spent in each chamber and in a one-inch diameter around the cylinders on each side (Anymaze). Social approach was calculated from behavior during the first stage taking the quotient of the time spent with the first stranger divided by the time spent in both sides overall. Social recognition was calculated from the second stage, using the time spent with the second stranger divided by the time spent in both sides overall. Statistical difference was evaluated with ANOVA and Student’s *t*-test.

Spontaneous Alternation: In a plastic Y maze with three 14 inch x 3 inch arms, mice were tested for working memory for 5 min. Mouse movement throughout the maze was monitored from live video recorded from above, noting arm entry order with experimenter blinded to group. For each set of three entries, the spontaneous alternation of those entries through all three arms was counted, and alternation percent calculated. Differences were evaluated with Student’s t-test.

Radial Arm Water Maze: Due to lack of resources, this task was not assessed in FGFR2 cKO mice. A 6-foot maze was filled with room temperature water and fitted with 6 dividers to create 6 radial arms., Mice were tested for spatial memory with distant visual spatial cues across two days with experimenter blinded to group [48]. Briefly, using visible and hidden platforms, mice were evaluated for working memory of platform location across 15 trials on day one. Day two of testing assessed consolidation of short-term memory using only the hidden platform across 15 trials. Working memory was calculated via arm entry errors and time to reach platform in three-trial blocks, with repeated measures ANOVA to evaluate group differences over time.

Elevated Plus Maze: In a Stoelting (Wood Dale, Illinois) Elevated Plus Maze, mice were tested for anxiety-like behavior for 5 min. Mouse movement throughout the maze was recorded using an overhead camera. The amount of time spent in each arm and the center of the maze was assessed (Anymaze). Differences were evaluated with repeated measures ANOVA across zones and with a t-test of the ratio of time spent in the open to closed zones.

### Gene expression

To evaluate the penetrance of Cre-mediated deletion of the *fgfr2* gene and other genes related to glial regulation of brain function, dorsal forebrain, hippocampus, or medial frontal cortex was dissected out from brains of animals which were neonatally exposed to tamoxifen through maternal injection. RNA was isolated using standard Trizol methods and concentration assessed (Nanodrop Spectrophotometer, Thermo Scientific). cDNA (Superscript III First Strand Synthesis Kit, Invitrogen) was used to evaluate relative gene expression to *β-actin* using TaqMan primers (*B-actin:* predeveloped; *vGat:* Mm00494138_m1; *vGlut1:* Mm00812886_m1; *Fgfr2:* Mm1269938_m1) and GeneAmp PCR Mastermix in a StepOne™ Instrument (Applied Biosystems).

### Immunocytochemistry

At least 10 days after completion of behavioral testing, animals were anesthetized and perfused (phosphate buffered saline (1X PBS), then 4% paraformaldehyde), and brain tissue was post-fixed, cryoprotected with a sucrose solution in 1X PBS and embedded in OCT compound, and cryostat (Leica, CM1900, Bannockburn, Illinois) sectioned at 50 µm thickness. Standard immunostaining methods were then used on free floating brain sections: blocking with 10% goat serum in 1XPBS, TritonX-100 and Tween20, incubation with primary antibodies GFAP (DAKO Z0334, rabbit, 1:500), VGLUT1 (EMD Millipore AB5905, guinea pig, 1:4000), PSD95 (Abcam Ab12093, goat, 1:500), gephyrin (SySy 147021, mouse, 1:500), VGAT (SySy 131002, rabbit, 1:1000), glutamine synthetase (EDM Millipore MAB302, mouse, 1:500) washing the primary antibodies 3x in 1XPBS, incubation with Alexa dye-conjugated secondary antibodies, anti guinea-pig or anti mouse Alexa 594, anti-rabbit or anti-goat Alexa 488 (1:500; Molecular Probes), and coverslipping using mounting medium with DAPI (Vector Laboratories, #H-1200).

### Stereology

Glutamine synthetase+ cells in coronal tissue sections were measured using fluorescent microscopy with a Zeiss Axiolmager M2 microscope. Stereological estimates of hippocampus and medial frontal cortex (mFC) cell densities were calculated using optical fractionator approach and unbiased counting rules with 3-dimensional 150 × 100 × 10 μm counting frames, on a 450 × 450 μm grid for mFC and 600 × 600 μm grid for hippocampal CA, using a 40× objective lens with experimenter blinded to group (Stereoinvestigator; MBF Biosciences). Stereological counting to determine cell density, displayed as means and standard errors of the mean, was performed in 3–8 serial coronal sections (every 10th section) of the adult mFC and the hippocampus as previously described [49, 50].

### Astrocyte morphology

Astrocytes, GFAP + cells with well-delineated cell bodies and branches, in the mFC and hippocampus were randomly selected for morphology reconstruction. Ten z-stacks per cell were acquired at 100x and traced in each experimental group (Control and FGFR2 nKO) by using Neurolucida 11.03 (MBF Bioscience, Williston, VT USA) The coordinate files obtained by the 3D reconstruction were analysed in Neuroexplorer [51].

### Punctal assessment

VGLUT1, PSD95, and colocalized puncta as well as VGAT, Gephyrin, and colocalized puncta were assessed by imaging the adult mFC of the FGFR2 nKO with a Zeiss Axioimager M2 microscope equipped with ApoTome2. Four mice per group were examined. Eight z-stacks spanning the entire cortical layers were imaged at 40x with experimenter blinded to group. The ImageJ software (National Institute of Health) Puncta Analyzer plugin for the estimation of these puncta was used as previously described [52].

### Electron microscopy

We performed assessments of glial and synaptic structure as previously [53]. Electron microscopy photographs (16,300×) were used to first measure the perimeter of each neuronal profile analyzed, followed by determination of the amount of membrane covered by astrocytes with experimenter blinded to group. The results are reported as percentage of astrocyte coverage neuronal cell membrane. Synaptic boutons in direct contacts with the same neuronal profiles analyzed for glial coverage was determined as described in our earlier studies [53–55]. Synapse number is reported per 100 μm perikaryal membrane.

### Statistical methods

Data normality and variance was assessed with GraphPad Prism8 to select appropriate statistical tests. Graphs were made and two-tailed Student’s t-tests were performed with Microsoft Excel. ANOVA for repeated measures outcomes were performed with GraphPad Prism8. Outliers were excluded if they were >2 standard deviations from the mean.

## Results

Mice with conditional loss of FGFR2 starting in embryonic radial glia (cKO) have been previously described [1, 8]. In brief, the FGFR2 cKO resulted from recombination of the conditional *fgfr2*^*f*^ alleles with the *hGFAP-cre* transgene, where Cre is expressed in radial glia beginning at embryonic day 13.5, therefore affecting all their neuronal and glial progeny in regions where the *hGFAP-cre* transgene is expressed, primarily the forebrain and cerebellum. An 80–91% loss of *fgfr2* gene expression was previously found and a substantial reduction of FGFR2 protein level [1]. The adult astrocyte FGFR2 knock out (FGFR2 iKO) has also been previously described, with an 80% reduction in *fgfr2* gene expression, and was accomplished by recombination of the same *fgfr2*^*f*^ alleles with the *hGFAP-CreER*^*T2*^ transgene, where Cre was expressed in postnatal GFAP^+^ glial cells after tamoxifen injection in adulthood [8].

To knock out FGFR2 in astroglial cells in the neonatal period (FGFR2 nKO), the same mice carrying *fgfr2*^*f*^ alleles and the *hGFAP-CreER*^*T2*^ transgene received tamoxifen via the milk in the neonatal period by injecting the Cre negative dam with 1 mg tamoxifen once daily for 5 days. The reduction of *fgfr2* assessed by qRT-PCR in juvenile or adult cortex or hippocampus varied between 29% and 43% (Supplementary Table 1). Control animals used for the FGFR2 nKO and FGFR2 iKO lines were also injected with tamoxifen to control for the potential impact of this manipulation.

### Embryonic knock-out of FGFR2: Behavioral changes

**Fig. 1:**
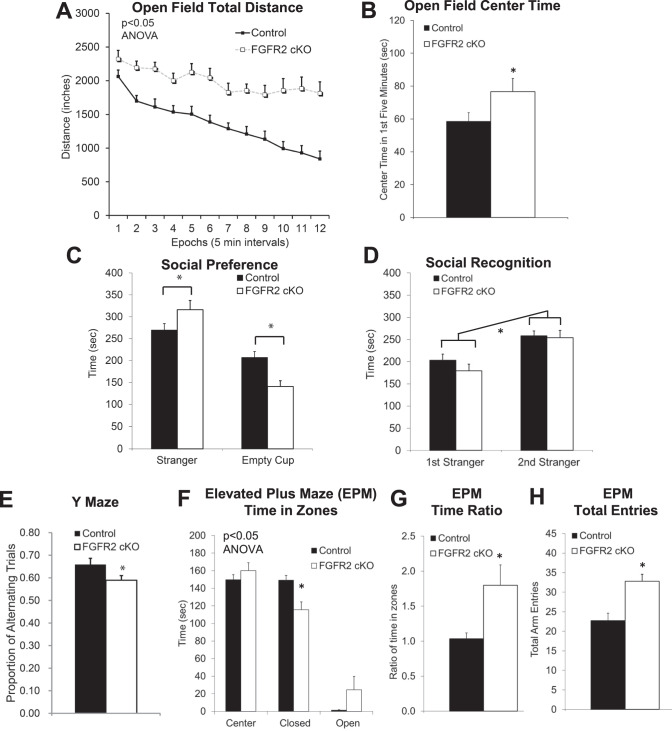
Adult male mice embryonically lacking FGFR2 driven by hGFAP-Cre (beginning by at least E13.5) showed locomotor hyperactivity, reduced anxiety-like behavior, increased sociability, and reduced working memory. **A** Persistently increased distance traveled in the open field in FGFR2 cKO mice. **B** Reduced anxiety-like behavior with open field increased time in the center in FGFR2 cKO mice. **C** Three chamber social task with social side compared with non-social side showed increased social preference in FGFR2 cKO mice. **D** Three chamber social task with familiar social side compared with novel social side showed no differences in social recognition. **E** Reduced working memory with less Y maze spontaneous alternation in FGFR2 cKO mice. **F** Reduced anxiety-like behavior with altered time spent in the closed and open arms of the EPM. **G** Reduced anxiety-like behavior with altered ratio of time in zones of the EPM. **H** Increase locomotor activity with increased overall entries into all arms of the EPM. *N* = 14,14; *p < 0.05 two-tailed Student’s *t*-tests or ANOVA. Means and SEM shown.

In FGFR2 cKO mice, multiple behavioral abnormalities were identified in addition to learning and memory deficits demonstrated previously [8]. Locomotor hyperactivity was found to be similar to that previously characterized in FGFR1 cKO mice (Fig. 1A; 55% greater; rmANOVA: F(1,26) = 20.17, *p* = 0.0001) [12]. Compared to their control littermates, FGFR2 cKO mice were more active in an open field, also spending more time in the center of the open field (Fig. 1B; *p* = 0.04)), a phenotype suggesting reduced anxiety-like behavior.

FGFR2 cKO mice also showed alterations in social approach. When tested on their social preference, they demonstrated a small but significantly higher preference than controls for interacting socially with a novel stranger versus spending time with a novel object (Fig. 1C; ANOVA interaction: F(1,55) = 12.24, *p* = 0.0009). This higher sociability was true in approach behavior in close proximity to the novel mouse or object (sociability index 32% increase: cKO: 0.77 vs controls: 0.58, *p* = 0.0003), as well as in larger chambers (sociability index 26% increase: cKO: 0.68 vs controls: 0.54, *p* = 0.002). This effect was not attributable to altered social recognition, as both cKO and control mice had similar interaction with a familiar versus a stranger mouse target (Fig. 1D; recognition indices: large chamber cKO: 0.60 vs controls: 0.53, *p* = 0.19; close proximity cKO: 0.64 vs controls: 0.61, *p* = 0.46).

Working memory was impaired in FGFR2 cKO mice (Fig. 1E, *p* = 0.048), as shown by a small but significant 10% lower spontaneous alternation in a Y maze.

Behavior on the elevated plus maze was also significantly different from control littermates overall (Fig. 1F, ANOVA interaction: F (2,78) = 7.991, *p* = 0.0007). Mice demonstrated a small effect on anxiety-like behavior, with less time spent in the closed arms of the maze (*p* = 0.02) and a lower ratio of time across the elevated plus maze zones (Fig. 1G; *p* = 0.03), but no difference in the ratio of entries across zones (data not show; *p* = 0.32). Performance on the elevated plus maze, a different environment than an open field, also confirmed the higher activity level of these mice regardless of context (Fig. 1H; *p* = 0.001).

### Neonatal knock-out of FGFR2: Behavior changes

**Fig. 2:**
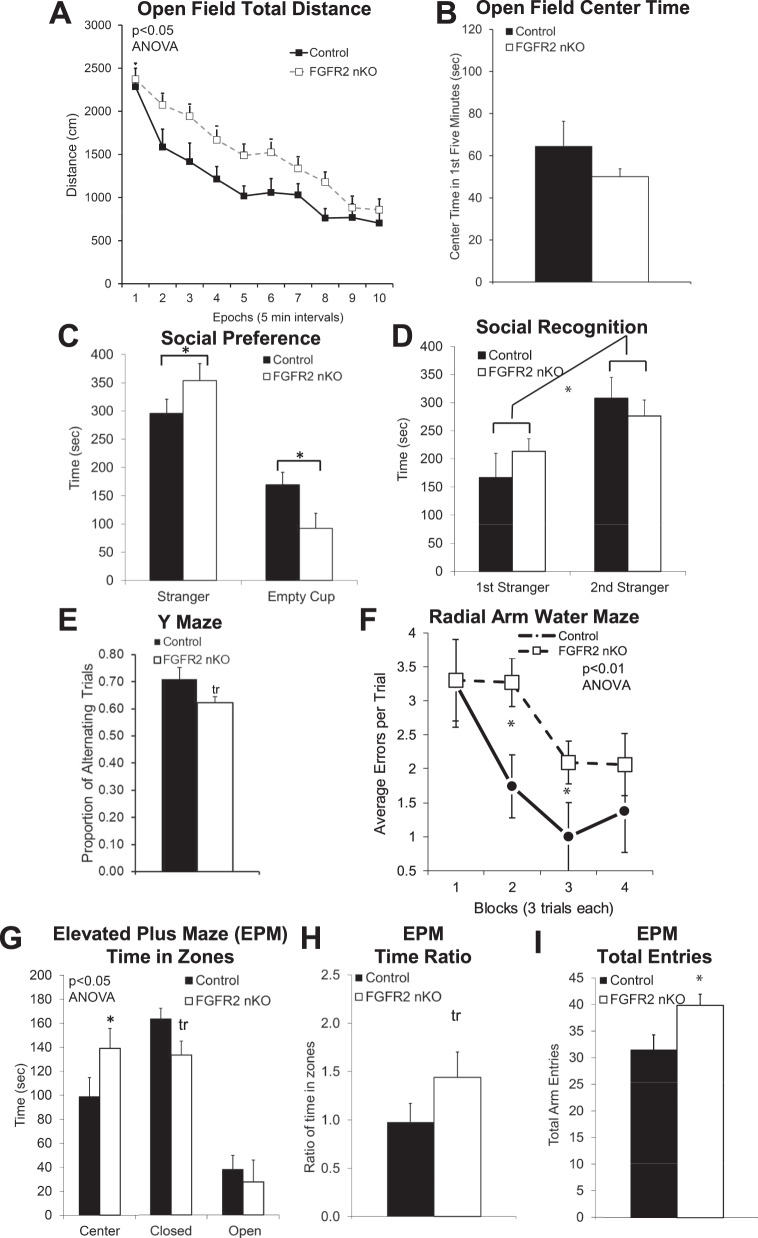
Adult male mice early postnatally lacking FGFR2 driven by GFAP- CreER^T2^ (induced with neonatal tamoxifen injections P1-7) showed locomotor hyperactivity, reduced anxiety-like behavior, increased sociability, and reduced working memory. **A** Persistently increased distance traveled in the open field in FGFR2 nKO mice. **B** No differences in anxiety-like behavior with open field time in the center. **C** Three chamber social task with social side compared with non-social side showed increased social preference in FGFR2 nKO mice. **D** Three chamber social task with familiar social side compared with novel social side showed no differences in social recognition. **E** Reduced working memory with less Y maze spontaneous alternation in FGFR2 nKO mice. **F** Reduced working memory with increased errors in the Radial Arm Water Maze training trials in FGFR2 nKO mice. **G** Reduced anxiety-like behavior with altered time spent in the closed and center arms of the EPM. **H** Reduced anxiety-like behavior with altered ratio of time in zones of the EPM. **I** Increased locomotor activity with increased overall entries into all arms of the EPM. *N* = 10,10; *p < 0.05 two-tailed Student’s *t*-tests or ANOVA. Means and SEM shown.

The behavior of FGFR2 nKO mice, lacking FGFR2 signaling only in GFAP^+^ astroglial cells beginning in the neonatal period, was characterized on the same tasks described above (Fig. 2A–H) as well as further characterization of working memory with a radial arm water maze. Compared to Cre- negative littermates with the same tamoxifen exposure, FGFR2 nKO animals showed a small but significant 32% increase in locomotor activity in an open field but no increase in time in the center (Fig. 2A, B; rmANOVA: F (1,15) = 5.542 *p* = 0.03; center time *p* = 0.97). We also examined activity level of FGFR2 nKO mice compared to an additional control group—a small sample of vehicle/oil injected Cre+ controls. Activity of FGFR2 nKO mice also trended increased by this comparison (rmANOVA *n* = 3 oil inj Cre+ vs *n* = 10 tam inj Cre + , rmANOVA: F (1, 11) = 4.574, *p* = 0.056, Supplementary Fig. 1A), and tamoxifen injection itself compared to oil in Cre- mice did not alter open field behavior.

FGFR2 nKO mice also showed the same small increased preference for social interaction indicating higher sociability (Fig. 2C: ANOVA interaction: F (2,28) = 3.343, *p* = 0.04; sociability index: large chamber 31% increase: nKO: 0.76 vs controls: 0.58, *p* = 0.02; close proximity 21% increase: nKO: 0.75 vs controls: 0.62, *p* = 0.11) without a deficit in social recognition (social recognition index: large chamber: nKO: 0.64 vs controls: 0.57, *p* = 0.49; close proximity: nKO: 0.70 vs controls: 0.66, *p* = 0.64) (Fig. 2C, D).

Working memory on the Y maze was trend deficient in FGFR2 nKO mice to the same small extent (12%) as shown for FGFR2 cKO mice (Fig. 2E, *p* = 0.07). Working memory was further measured by performance on the radial arm water maze (Fig. 2F). This task also showed that working memory, as measured by errors during the training phase when animals must keep location information in working memory, was significantly impaired with small effect size in FGFR2 nKO mice (rmANOVA: F (2.725, 49.06) = 5.363, *p* = 0.0037).

Measures of anxiety-like behavior on the elevated plus maze was also shown to be altered in FGFR2 nKO mice in a comparable fashion to the FGFR2 cKO mice, with a small reduction in anxiety-like behavior: less time in the closed arms of the maze and more time in the center (Fig. 2G, ANOVA interaction: F (2,48) = 3.414, *p* = 0.04; *p* = 0.08, *p* = 0.048). FGFR2 nKO mice showed a trend higher ratio of time in the open to closed zones (Fig. 2H, *p* = 0.08), but no difference in the ratio of entries in the zones (data not shown; *p* = 0.34). We also examined these differences in the vehicle/oil injected animals and found that tamoxifen injection which induced the FGFR2 nKO was needed to see this effect (Supplementary Fig. 1B). Just as seen in FGFR2 cKO mice, levels of activity in FGFR2 nKO mice were confirmed to be increased to a small extent on the EPM compared to controls (Fig. 2I, *p* = 0.01).

In summary, the induced loss of FGFR2 in neonatal astrocytes resulted in many alterations on the same behaviors as those noted here in the FGFR2 cKO mice in which the FGFR loss starts in radial glial cells in the embryonic period—social preference behavior, spontaneous alternation rate, and elevated plus maze closed arm time and total entries were changed to similar extents in both types of FGFR2 deficit mice. In FGFR2 nKO mice, open field activity and elevated plus maze time ratio were increased as in FGFR2 cKO animals but not to the same extent; additionally, open field center time was not increased in FGFR2 nKO mice unlike FGFR2 cKO animals.

### Adult Knock-Out of FGFR2: Behavior Changes

**Fig. 3:**
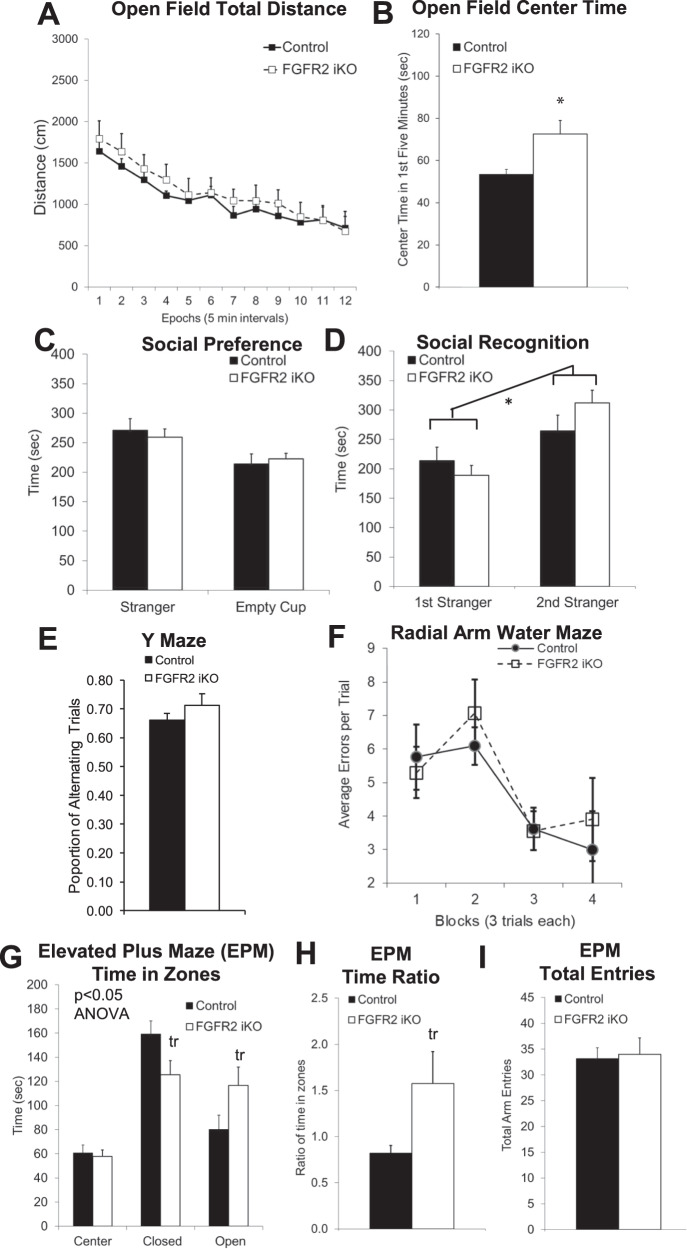
Adult male mice lacking FGFR2 in adulthood driven by GFAP- CreER^T2^ (induced with adult tamoxifen injections P56-60) showed only reduced anxiety-like behavior. **A** No difference in distance traveled in the open field. **B** Reduced anxiety-like behavior with open field increased time in the center in FGFR2 iKO mice. **C**, **D** Three chamber social task showed no difference in social preference or social recognition. **E** No difference in working memory with Y maze spontaneous alternation. **F** No difference in working memory with errors on Radial Arm Water Maze training trials. **G** Reduced anxiety-like behavior with altered time spent in the closed and open arms of the EPM. **H** Reduced anxiety-like behavior with altered ratio of time in zones of the EPM. **I** No difference in locomotor activity with overall entries into all arms of the EPM. *N* = 7,10; *p < 0.05 two-tailed Student’s t-tests or ANOVA. Means and SEM shown.

In contrast to the comparable behavioral alterations of FGFR2 cKO and nKO mouse models, FGFR2 iKO mice, lacking FGFR2 signaling only in GFAP^+^ glial cells beginning in adulthood, showed no alterations of locomotor activity on the open field, working memory on the Y maze or the radial arm water maze, or social preference (Fig. 3A, C, D, E, F). The similar behavior of Cre- control and Cre+ FGFR2 iKO on many behaviors validated that the Cre transgene was not, itself, a source of behavioral differences. We verified that Cre- control mice injected with tamoxifen in adulthood did not differ from Cre- control mice injected with tamoxifen in the neonatal period when considering locomotor activity on the open field, working memory on the Y maze, or social preference (Supplementary Fig. 2A–C).

Differences of FGFR2 iKO mice from wild type littermates were found only for anxiety-like behavior. On the elevated plus maze (Fig. 3G, ANOVA interaction: F (2, 36) = 5.260, *p* = 0.0099) these FGFR2 iKO mice demonstrated trend less time spent in the closed arms (*p* = 0.05), more time in the open arms (*p* = 0.08), and higher ratio of time in the open to closed zones (Fig. 3H; *p* = 0.07), indicative of less anxiety-like behavior, but no differences in overall activity level (Fig. 3I) or in the ratio of entries in the zones (data not shown; *p* = 0.23). There was no effect of adult oil injection itself on elevated plus maze performance (Supplementary Fig. 1C), confirming that the adult FGFR2 iKO with tamoxifen was needed for this effect. This effect was clear despite the increased open arm time in Cre- control mice injected with tamoxifen in adulthood (Supplementary Fig. 2D). Decreased anxiety-like behavior was also demonstrated by increased time spent in the center of the open field (Fig. 3B, *p* = 0.02).

### Neurobiological findings

Given the multiple behavioral abnormalities induced when FGFR2 was lacking only beginning in neonatal life in primarily astrocytes, we performed pilot investigations of their neurobiology focused mainly on hippocampus as a major region implicated in regulation of the behaviors assessed here. We first assessed GFAP + astrocyte density in the hippocampus of FGFR2 nKO mice, finding no differences (*n* = 3,3; *p* = 0.36, control = 8.45 ± 0.97 × 10^−6^, FGFR2 nKO=10.14 ± 1.37 × 10^−6^ cells/µm^3^). The volume of the hippocampus was also unchanged (*n* = 3,3, *p* = 0.86, control = 3.1 ± 0.6 mm^3^, FGFR2 nKO = 3.2 ± 0.6 mm^3^). In addition, the most broadly affected model used here, FGFR2 cKO mice, showed no deficit in GFAP + cell density either (*n* = 3,3; *p* = 0.32, control = 9.26 ± 1.05 × 10^−6^, FGFR2 cKO = 8.44 ± 1.69 × 10^−6^ cells/µm^3^). This suggested that astrocyte numbers themselves were intact regardless of early loss of FGFR2.

**Fig. 4:**
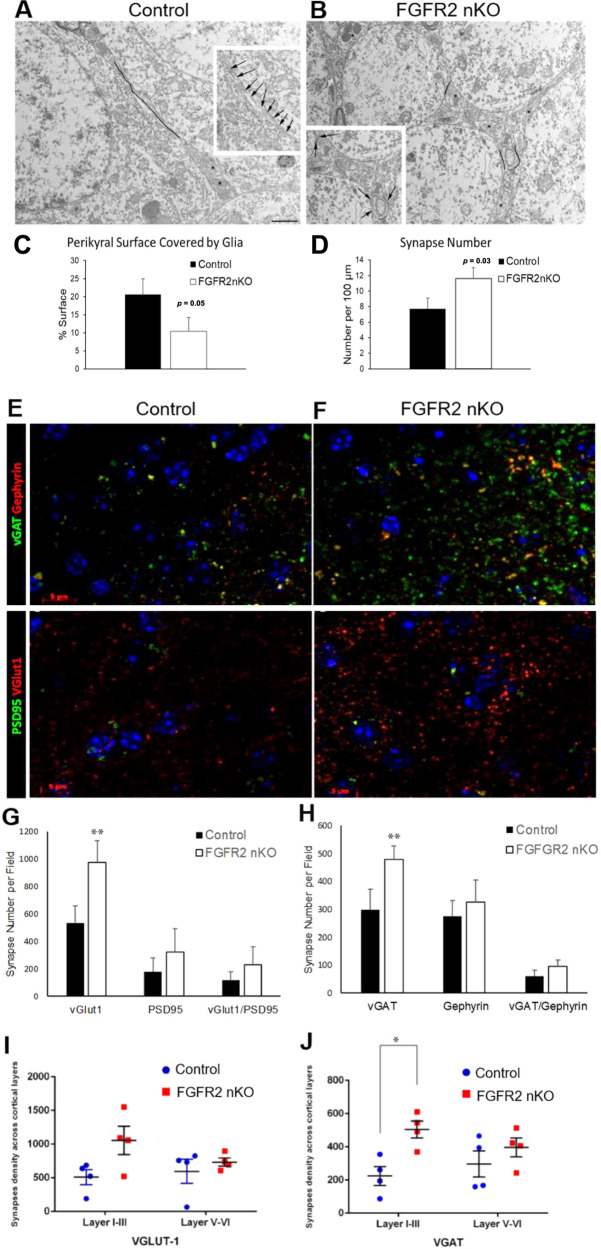
Adult male mice early postnatally lacking FGFR driven by GFAP- CreER^T2^ (induced with neonatal tamoxifen injections P1-7) show reduced astrocyte processes in neuropil by electron microscopy. **A**–**D** Less glial coverage of perikaryal membrane (black lines) and more synapse number on the same membrane (asterisks) in FGFR2 nKO compared to control littermate mice in the hippocampus CA3 region (30 cells per genotype). *N* = 3,3. These mice also show increased density of pre-synaptic proteins assessed by immunofluorescence. **E**–**H** Greater number of puncta of vGLUT1 and vGAT per field of analysis in lateral cortex. **I**, **J** Greater number of vGAT puncta in cortical layers I-III when evaluated separately from cortical layers IV-VI. *N* = 4,4; *p < 0.05, **p < 0.01 two-tailed Student’s t-tests or ANOVA. Means and SEM shown.

To gain insights into astrocyte morphology after early postnatal loss of FGFR2 in astroglia, we performed electron microscopy (EM) analyses of the hippocampus in FGFR2 nKO mice. The analysis of the astrocytic coverage of the cell membrane of neurons in the principal cell layer of the CA3 region of the hippocampus showed reduced coverage by almost half (Fig. 4A–C). Of note, this measure of astrocyte-neuron contact was coupled to increased number of synapses on the same cells (Fig. 4D).

The loss of FGFR2 signaling did influence the expression of glutamine synthetase (GS), a metabolic component of glial cells which is regulated by glutamate transport and synaptic activity. In FGFR2 nKO, the density of glutamine synthetase positive cells, as measured by stereological counts in adult brains, trended increased in hippocampus and was also increased to a greater extent in cerebral cortex of FGFR2 nKO mice (Table 1). Regardless of these changes, gross astrocyte morphology in hippocampus was qualitatively normal (Supplementary Fig. 3A). Increased glutamine synthetase expression was also found in the hippocampus after embryonic knock out of FGFR2 (FGFR2 cKO) which had similar behavioral alterations to nKO mice and also lacked FGFR2 in astrocytes from early developmental stages. However, glutamine synthetase change was not seen in FGFR2 iKO mice (Table 1).

**Table 1:** Glutamine Synthetase+ cell density differences with reduction of FGFR2 signaling.

	Control Mean ± SEM cells x 10^−5^/μm^3^	Experimental Mean ± SEM cells x 10^−5^/μm^3^	Difference	*p*-value
FGFR2 nKO Medial Frontal Cortex	0.23 ± 0.01 (*n* = 3)	0.47 ± 0.05 (*n* = 3)	↑104%	0.01*
FGFR2 nKO Hippocampus	1.20 ± 0.15 (*n* = 3)	1.99 ± 0.43 (*n* = 3)	↑68%	0.07^¥^
FGFR2 cKO Hippocampus	1.55 ± 0.06 (*n* = 4)	2.05 ± 0.13 (*n* = 6)	↑30%	0.02*
FGFR iKO Hippocampus	1.77 ± 0.19 (*n* = 3)	2.09 ± 0.40 (*n* = 3)	↑18%	0.55

^*^p < 0.05, ¥-trending significance, 0.05 ≤ p < 0.10.

With the greater change in astrocyte glutamine synthetase expression in the cortex of the FGFR2 nKO mice, we examined cortical density of synaptic proteins in adult FGFR2 nKO mice, as assessed by immunohistochemical puncta analysis. FGFR2 nKO mice were found to have increased puncta density after immunostaining for both the GABA neurotransmitter release protein, vGAT, and the glutamate neurotransmitter release protein, vGLUT1 (Fig. 4E–J). In contrast, the density of post-synaptic protein puncta (gephyrin and PSD95) or co-localized pre- and post-synaptic protein densities were unchanged (data not shown). The vGAT and vGLUT1 punctal increases were localized to upper cortical layers I-III (Fig. 4I, J).

Lastly, we examined gene expression of these same synaptic proteins in the hippocampus of another cohort of FGFR2 nKO mice and controls (*n* = 8,6). GABA transporter *vGat* (by RT-qPCR) was increased and synaptic glutamate transporter *vGlut1* trended increased in the juvenile hippocampus (*n* = 8,5; *vGat*: *p* < 0.005, control = 1.00 ± 0.07, FGFR2 nKO = 3.62 ± 0.91; *vGlut1*: *p* = 0.09 control = 1.00 ± 0.12, FGFR2 nKO = 3.70 ± 1.86). These findings suggest that early postnatal glial FGFR2 loss in the dorsal forebrain induces a decrease in astrocyte-neuron contacts and potentially an increase in neuronal synaptic contacts, as shown by EM and level of presynaptic marker expression, particularly in hippocampus.

## Discussion

We have demonstrated a distinct behavioral triad of hyperactivity, working memory deficits, and increased sociability in mice lacking FGFR2 in astroglial cells, only when that loss begins by at least the neonatal period of development. In concomitance with this behavior, the loss of FGFR2 in neonatal astroglial cells results in data suggesting decreased astroglia-neuron membrane appositions as well as increased neuronal synapses and their signaling proteins, as shown by EM and puncta density of presynaptic vesicular proteins. We further show that expression of GS, a critical astrocytic protein for both glutamatergic and GABAergic synaptic function [56, 57], is increased in astroglia. We hypothesize that the increase in GS immunostaining likely represents a functional aftereffect of increased neuronal signaling, informed by others’ findings that GS expression is influenced by neuronal activity [56, 58]. Distinct from these phenotypes, we found that a small decrease in anxiety-like behavior was present in animals with induced loss of FGFR2 in GFAP + cells at all developmental time periods, even when induced only in adulthood. This suggests that hyperactivity, working memory deficits, and increased sociability might be related to the developmental roles of FGFR2 in astrocytes in the neonatal period, whereas the modest decrease in anxiety-like behavior might reflect a continuous, ongoing role of FGFR2 in the functioning of astroglial cells as we previously demonstrated for short term memory [8].

Fibroblast growth factor signaling in the brain has previously been investigated for its role in embryonic patterning and regulation of neurogenesis [19] or, in adulthood, for its implications in behavioral alterations, such as anxiety-like behavior and learning [8, 10, 13]. Because hyperactivity, working memory deficits, and increased sociability were present here when FGFR2 was lacking from the neonatal period onward but not when the loss was induced in adulthood, these results support a new line of thinking, implicating fibroblast growth factor signaling in the early postnatal brain [59] and further suggest that these processes may affect the risk for behavioral disorders [38]. Convergent data on knock out of early postnatal FGF22, a ligand partner of FGFR2, affecting anhedonia supports this idea [18]. These findings together support the notion of “sensitive periods” in development, implying a crucial role of FGFR2 signaling in the perinatal/juvenile period.

The three cohorts here with FGFR2 loss at different time points, as well as in different subsets of cells, showed some similarity and some difference. All three FGFR2 KO lines which had in common their lack of FGFR2 in astroglial cells in adulthood showed small reductions in anxiety-like behavior in the elevated plus maze, as assessed by small shifts in time from closed to open zones; open arm time, the metric most robustly associated with anxiety-like constructs in the literature, was not always increased and EPM findings were only trending at times. These findings of small effect may reflect FGFR2’s mixed roles with multiple ligands (FGFs and other molecules which bind to FGFR2) [60, 61] in regulating anxiety-like behavior [10, 62, 63]. The clearest findings were in the adult-induced FGFR2 knock-out mice (FGFR2 iKO); this suggests that the reduced anxiety-like behavior found in embryonically-induced FGFR2 knock-out (FGFR2 cKO) and FGFR2 nKO mice may also be attenuated by the other roles FGFR2 plays in early development potentially underlying their other behavioral deficits. For example, greater locomotor hyperactivity in the FGFR2 cKO mice compared to FGFR2 nKO and FGFR2 iKO may interact with their anxiety-like behavior regulation in complex ways.

More distinct differences between these three FGFR2 KO lines included the presence of the deficits in hyperactivity, sociability, and working memory in only FGR2 cKO and FGFR2 nKO mice. Analysis of results from vehicle/oil injected mice and comparing mice tamoxifen-injected at different ages provided reassurance that differences were due to the knock out of *fgfr*. The 25–30% increase in the social preference index was similar in both these mouse lines and involved increased time spent with a novel stranger mouse relative to time spent with an empty social interaction cup. This may reflect a deficit in typical down-regulation of social approach over time or an increased drive for social reward. While models for the study of neuropsychiatric disorders commonly demonstrate decreased sociability [64], increased rodent social interaction on a variety of tasks has also been demonstrated in a number of studies with different genetic backgrounds relevant to autism spectrum disorder (ASD), schizophrenia, intellectual disability, mood disorders, and attention deficit hyperactivity disorder (ADHD) [64–71]. Other studies have demonstrated heritable patterns of increased social interaction in monkeys which has also been described as social impulsivity [72]. Directionality of behavior on non-human animal social tests cannot be directly compared to human social interactions, although may generally inform the understanding of the development of neural systems underlying social behavior.

Further behavior similarities arose when FGFR2 loss began early in development. While working memory in FGFR2 cKO mice was only tested with the Y maze, this Y maze deficit was very similar to that in the FGFR2 nKO mice (small 10% and 12% deficits respectively) which also showed a radial arm water maze working memory deficit. These deficits were distinct from the short and long term memory deficits previously identified with FGFR2 iKO and cKO mice respectively [8], but findings here were similar to other working memory deficits found with targeted astrocyte dysfunction [73, 74] and models for the study of ADHD [75]. Lastly, hyperactivity levels were much greater in FGFR2 cKO compared to FGFR2 nKO mice (55% vs 32%), suggesting that the earlier and greater loss of FGFR2 expression including in all dorsal forebrain neurons and glia may underlie this outcome.

The importance of glial cells during early developmental time periods has been suggested by other lines of research. Glial proliferation occurs predominantly in the last few embryonic days and first few postnatal weeks of mouse development which also may be a critical time period for establishing these cells’ own later functioning. Although FGF signaling regulates glial proliferation and fate [31, 76], astrocyte density was not reduced by the early loss of FGFR2 in this study. This suggests that this process is redundant between different FGFRs and/or is regulated by non-FGFR2 mechanisms. In the neonatally-induced loss of FGFR2 in GFAP expressing cells, impacts on behavior may be from alterations in more differentiated glial cells. We cannot exclude that loss of FGFR2 in a small number of neural stem cells and their neuronal progeny targeted by *GFAP*-*creER*^T2^ also plays a role in behavioral impacts in the tamoxifen-induced lines. However, regardless of possible inclusion of neuronal FGFR2 signaling in these mechanisms, there were definitive impacts on astrocytes. Our neurobiological data suggest that the deficiency of FGFR2 has an impact on astrocyte differentiation and maturation, processes that occur in the early postnatal period in mouse brain, and which may result in abnormal astrocyte ultrastructure. Astrocyte maturation is regulated by multiple FGF ligands, including changes in protein levels that occur as astrocytes mature: for example, upregulation of the glutamate transporter, GLT-1, and downregulation of GFAP [31]. Our data suggests that FGFR2 may be an important signaling partner for these FGF ligands in these processes.

Early postnatal astrocytes are in a distinct phase of differentiation; [77] as astrocytes develop, cellular extensions are made to promote astroglia-neuron interactions [78], the extent of which we found to be deficient at the ultrastructural level with FGFR2 knockout, similar to findings in drosophila lacking the FGF receptor, Heartless [79]. Decreased FGF signaling reduced astrocytic coverage of neuronal membranes, with synaptic processes increased in these same glial-neuronal couplets. Astrocyte-neuron contacts may in turn regulate the number of neuronal synapses during the early postnatal period by competitive processes or by affecting pruning of synapses, a process that is time-dependent [80, 81] and regulated by astrocytes [80, 82]. Synaptic pruning during these sensitive periods of development allows for experience to shape brain development [38, 39]. In sum, the present study suggests a possible competitive antagonism between astrocyte/neurons at synapses which may be important in regulation of neuronal signaling and synaptic pruning in early postnatal development. These data suggest intriguing mechanisms that should be assessed in future studies.

Given that punctal density of vesicular proteins was increased in neonatally-induced FGFR2 knockout mice when assessed by immunocytochemistry, a disruption of astrocyte regulation of neuronal signaling is likely. Increased glial metabolism, as shown through increased glutamine synthetase positive cells, may reflect a response to a higher level of neuronal signaling from increased vesicular proteins. A similar reduction in FGFR2 signaling in glial cells just a few days later in juvenile brain (postnatal day 8) decreased vesicular proteins [9] suggesting a dynamic role of this receptor with shifting impacts on neuronal structure and functioning as synapse formation, elimination, and maintenance occurs in the developing dorsal forebrain.

Astrocyte functioning has also been implicated in psychiatric disorders [83–85], with a main focus in adult psychopathology. The role of astrocytes in common psychiatric disorders of childhood, in which clinical impairment and pathophysiological mechanisms clearly begin earlyis potentially important given the early timing of astrocyte maturation [41, 86, 87]. Glial functioning may be altered in patients with ASD [88–90] and ADHD [91, 92]. Clinical investigations in ADHD show decreased cortical surface area during childhood [93], which has implications for many aspects of neurobiology beyond dopamine and norepinephrine signaling that are targeted by current treatments. Proton magnetic resonance spectroscopy studies have implicated glutamate-glutamine metabolism in ADHD, which is highly dependent on astrocyte functioning [94]. Especially intriguing, as shown by the results here, is the potential role in childhood psychopathology of astrocytes in their early postnatal phases of differentiation. Astrocyte differentiation could be affected by early postnatal developmental insults [58, 95] but could also be a mechanism by which inherited risk for ADHD and other disorders is manifested.

The range of behavioral alterations manifested by the glial-targeted FGFR2 mice resembles that which individuals with ADHD-combined type display; the diagnostic criteria include increased locomotor activity, poor attention—relevant to working memory deficits— and impulsivity which leads to dysregulated increased social interaction. Some other models for the study of ADHD consistently show all of these impacts, although social approach is not a task typically assessed [75]. Because of the importance of social functioning for children’s success, we suggest this may be an important consideration for future studies. This and other studies suggest the possibility that diagnostic and treatment options for ADHD could be developed to incorporate glial functioning as a target generally or specifically with nanomedicine technological advances [96]. Treatments with great efficacy for ADHD exist. However, because of the relatively common occurrence of the disorder [97], significant numbers of children, adolescents, and adults are not successfully treated and experience great impairments in their functioning. Advancements in understanding the neurobiology of ADHD has the potential to benefit many.

## Supplementary information

Supplemental Material
