# Supplementary material for: Neonatal loss of FGFR2 in astroglial cells affects locomotion, sociability, working memory, and glia-neuron interactions in mice
Source: Transl Psychiatry. 2023 Mar 11;13:89. doi: 10.1038/s41398-023-02372-y (PMC10008554; doi:10.1038/s41398-023-02372-y)
Supplement: Supplementary file 1 — Supplemental Material [file 41398_2023_2372_MOESM1_ESM.pdf]

Supplemental Fig 1

Additional Controls: **A,B.** Comparison of neonatal vehicle/oil injected FGFR2 Cre- and Cre+ control mice with neonatal tamoxifen injected FGFR2 Cre- and nKO Cre+ mice on two key behavioral tasks for which there were differences for Tam-induced FGFR2 nKO.

A. Increased activity in open field of Tam-injected Cre+ animals compared to Oil-injected Cre+ animals (rmANOVA n=3 oil inj Cre+ vs n=10 tam inj Cre+, rmANOVA: $F(1, 11) = 4.574$, $p=0.056$). Neonatal Tam Inj FGFR2 Cre- mice (n=10) and Neonatal Oil Inj Cre+ mice (n=5) included for demonstration of similarity to Neonatal Oil Inj FGFR2 Cre+ mice.
B. Time Ratio in Zones of Elevated Plus Maze. Difference of Cre- and Cre+ Tam Inj mice (n=10,10) shown for comparison to no difference in Cre- and Cre+ Oil Inj mice (n=3,5). (NS= No significant difference or trend)

C. Comparison of adult vehicle/oil injected FGFR2 Cre- and Cre+ control mice with adult tamoxifen injected FGFR2 Cre- and iKO Cre+ mice on the only behavioral task for which there was a difference for Tam-induced FGFR2 iKO. Time Ratio in Zones of the Elevated Plus Maze. Difference of Cre- and Cre+ Tam Inj mice (n=7,10) shown for comparison to no difference in Cre- and Cre+ Oil Inj mice (n=3,4) (NS= No significant difference or trend)

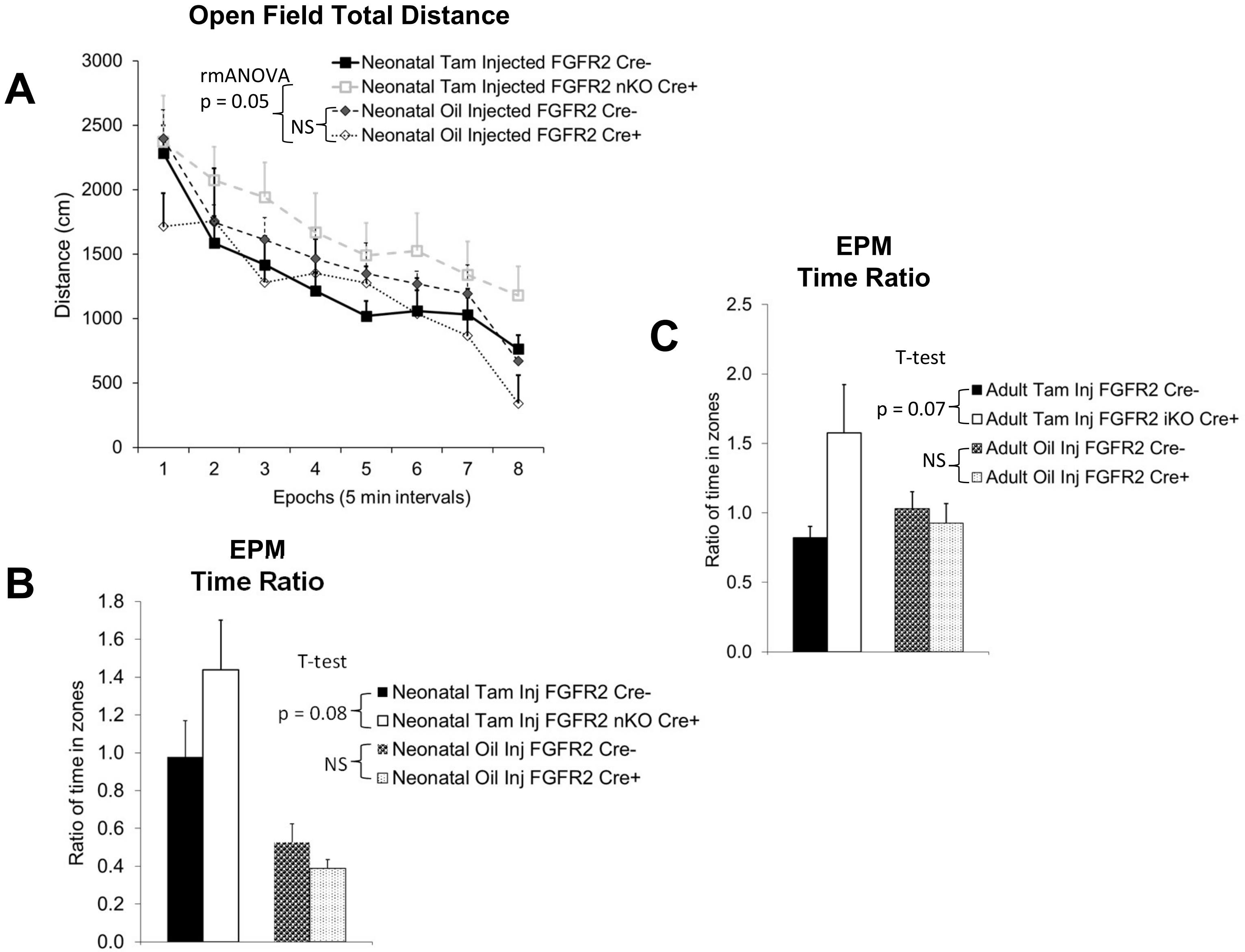

Supplemental Fig 2

Comparison of neonatal tamoxifen injected FGFR2 Cre- controls with adult tamoxifen injected FGFR2 Cre- controls (n=9 neonatal tam vs n=6 adult tam). A: No difference in Open Field Activity (rmANOVA: $F (1, 130) = 1.034$, $p=0.311$); B: No difference in Y-maze Spontaneous Alternation ($p=0.38$); C: No difference in Social Preference (two-way ANOVA interaction: $F (1, 26) = 1.726$, $p=0.2004$); D. Increased time in Open Arms of Elevated Plus Maze in adult tamoxifen injected FGFR2 Cre- Controls ($p=0.012$).

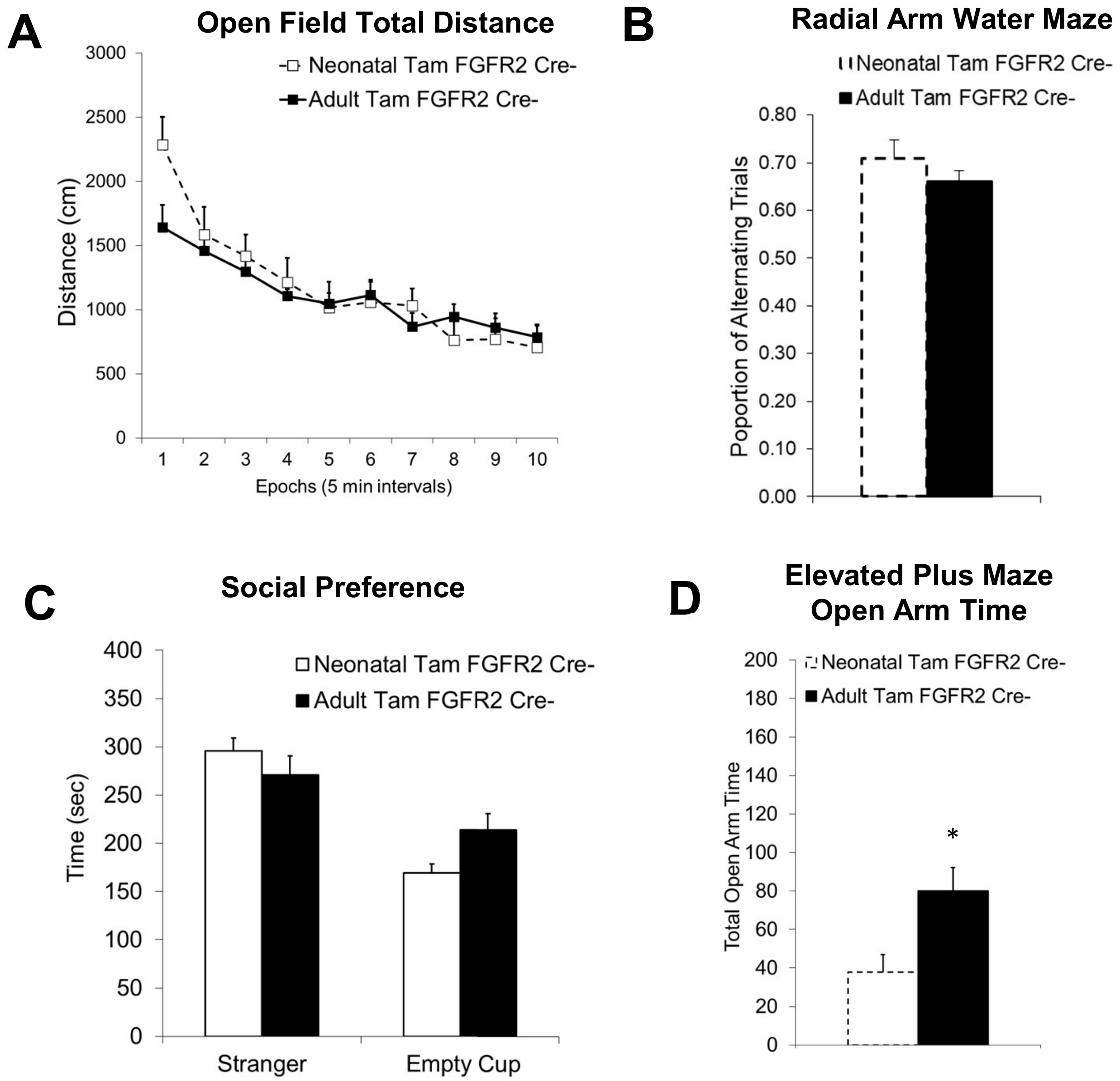

Supplemental Fig 3

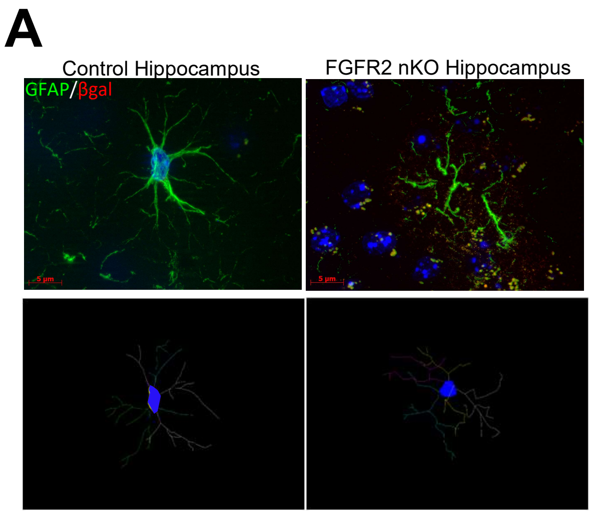

Visualization of overall normal astrocyte structure in control and FGFR2 nKO mice

Supplemental Table 1. Quantification of FGFR2 knock out with neonatal tamoxifen induction of *hGFAP-CreER*^{T2}

	**Control *Fgfr2* Gene Expression Mean ± SEM**	**FGFR2 nKO *Ffgr2* Gene Expression Mean ± SEM**	**Difference**	**P value Difference**
**Juvenile Cortex**	1.00 ±0.07 (n=8)	0.71 ± 0.07 (n=6)	↓29%	0.08¥
**Juvenile Hippocampus**	1.00 ±0.08 (n=8)	0.61 ± 0.04 (n=6)	↓39%	0.03*
**Adult Anterior Cortex**	0.87 ±0.10 (n=3)	0.49 ± 0.04 (n=3)	↓43%	0.03*
**Adult Posterior Cortex**	1.21 ±0.11 (n=3)	0.69 ± 0. (n=3)	↓43%	0.01*
**Adult Hippocampus**	0.74 ±0.14 (n=3)	0.5 ±0.07 (n=3)	↓33%	0.18

* significance $p<0.05$; ¥-trending significance, $0.05 \leq p<0.15$
